# The co-inhibitory receptor TIGIT regulates NK cell function and is upregulated in human intrahepatic CD56^bright^ NK cells

**DOI:** 10.3389/fimmu.2023.1117320

**Published:** 2023-02-09

**Authors:** Annerose E. Ziegler, Pia Fittje, Luisa M. Müller, Annika E. Ahrenstorf, Kerri Hagemann, Sven H. Hagen, Leonard U. Hess, Annika Niehrs, Tobias Poch, Gevitha Ravichandran, Sebastian M. Löbl, Benedetta Padoan, Sébastien Brias, Jana Hennesen, Myrtille Richard, Laura Richert, Sven Peine, Karl J. Oldhafer, Lutz Fischer, Christoph Schramm, Glòria Martrus, Madeleine J. Bunders, Marcus Altfeld, Sebastian Lunemann

**Affiliations:** ^1^ Research Department Virus Immunology, Leibniz Institute of Virology, Hamburg, Germany; ^2^ I. Department of Medicine, University Medical Center Hamburg-Eppendorf, Hamburg, Germany; ^3^ Institute of Experimental Immunology and Hepatology, University Medical Center Hamburg-Eppendorf, Hamburg, Germany; ^4^ University of Bordeaux, Institut National de la Santé et de la Recherche Médicale, Bordeaux Population Health Research Center, UMR1219 and Inria, Team Statistics in systems biology and translationnal medicine (SISTM), Bordeaux, France; ^5^ Institute for Transfusion Medicine, University Medical Center Hamburg-Eppendorf, Hamburg, Germany; ^6^ Department of General and Abdominal Surgery, Asklepios Hospital Barmbek, Semmelweis University of Medicine, Hamburg, Germany; ^7^ Department of Visceral Transplant Surgery, University Medical Center Hamburg-Eppendorf, Hamburg, Germany; ^8^ Martin Zeitz Centre for Rare Diseases, University Medical Center Hamburg-Eppendorf, Hamburg, Germany; ^9^ III. Department of Medicine, University Medical Center Hamburg-Eppendorf, Hamburg, Germany

**Keywords:** intrahepatic NK cells, liver organoids, single-cell mRNA analysis, immune tolerance, tissue homeostasis, TIGIT, DNAM-1, PVR/CD155

## Abstract

The crosstalk between NK cells and their surrounding environment is enabled through activating and inhibitory receptors, which tightly control NK cell activity. The co-inhibitory receptor TIGIT decreases NK cell cytotoxicity and is involved in NK cell exhaustion, but has also been associated with liver regeneration, highlighting that the contribution of human intrahepatic CD56^bright^ NK cells in regulating tissue homeostasis remains incompletely understood. A targeted single-cell mRNA analysis revealed distinct transcriptional differences between matched human peripheral blood and intrahepatic CD56^bright^ NK cells. Multiparameter flow cytometry identified a cluster of intrahepatic NK cells with overlapping high expression of CD56, CD69, CXCR6, TIGIT and CD96. Intrahepatic CD56^bright^ NK cells also expressed significantly higher protein surface levels of TIGIT, and significantly lower levels of DNAM-1 compared to matched peripheral blood CD56^bright^ NK cells. TIGIT^+^ CD56^bright^ NK cells showed diminished degranulation and TNF-α production following stimulation. Co-incubation of peripheral blood CD56^bright^ NK cells with human hepatoma cells or primary human hepatocyte organoids resulted in migration of NK cells into hepatocyte organoids and upregulation of TIGIT and downregulation of DNAM-1 expression, in line with the phenotype of intrahepatic CD56^bright^ NK cells. Intrahepatic CD56^bright^ NK cells represent a transcriptionally, phenotypically, and functionally distinct population of NK cells that expresses higher levels of TIGIT and lower levels of DNAM-1 than matched peripheral blood CD56^bright^ NK cells. Increased expression of inhibitory receptors by NK cells within the liver environment can contribute to tissue homeostasis and reduction of liver inflammation.

## 1 Introduction

Natural Killer (NK) cells are innate lymphocytes that not only play an essential role in the defense against pathogens and tumors but are also involved in modulating immune homeostasis and remodeling of tissues (1–5). Adequate regulation of immune surveillance and function in liver tissues is critical (6), as the liver is exposed to various antigens and metabolites derived from the gastrointestinal tract (7). NK cells represent up to 40% of all intrahepatic lymphocytes, and their role in maintaining immune homeostasis in the liver has received increasing attention (7–9). Intrahepatic NK cells have been associated with a broad spectrum of NK cell functions, ranging from exerting cytotoxicity, producing cytokines to mediating antigen-specific contact hypersensitivity responses (10–13). Based on the expression of the surface markers CD56 and CD16, NK cells can be divided into a CD56^bright^ and a CD56^dim^ subset (14). While CD56^dim^ NK cells predominate in the peripheral blood (pb) (15–17), CD56^bright^ NK cells are the main population of liver-resident (lr) NK cells, as defined by their expression of specific markers regulating liver residency of NK cells in humans, including CXCR6, CD69, CCR5 and CD49a (18–21). CXCR6^+^ lrNK cells express high levels of the transcription factor Eomes and low levels of Tbet (16, 22), and subsets of lrNK cells also express the transcription factors Hobit (15) and promyelocytic leukemia zinc finger protein (PLZF) (23). These distinct profiles in the expression of transcription factors have been suggested as potential drivers of tissue-specific regulation of NK cell function. Despite differences between CD56^bright^ lrNK cells and in particular CD56^dim^ as well as CD56^bright^ pbNK cells in the expression of transcription factors, the consequences for tissue-specific adaption of NK cell functions are insufficiently understood (18, 20, 23, 24).

NK cell activity is tightly controlled through a plethora of receptors, balancing activating and inhibitory signals. These highly diverse receptors enable the crosstalk between NK cells and their surrounding environment (24, 25). The T cell immunoglobulin and ITIM domain (TIGIT) is a co-inhibitory receptor that was first described to be expressed on T cells and NK cells (26–28). TIGIT is part of the immunoglobulin-superfamily and interacts with ligands of the PVR family, nectin and nectin-like family (26). TIGIT binds CD155 (poliovirus receptor; PVR), CD112 (PVRL2) CD113 (PVRL3), and Nectin4 (PVRL4), although the interaction of TIGIT with PVR showed to have the highest affinity (27, 29). PVR is abundantly expressed on antigen-presenting cells (APC) such as dendritic cells, as well as on T cells and in particular on tumor cells (30–32). Three other receptors, namely CD226 (DNAM-1), CD96 (TACTILE) and KIR2DL5 also interact with PVR, but with lower binding affinity, displaying diverse functions (27, 33–36). Binding of DNAM-1 promotes anti-tumor responses, enhances NK cell cytotoxicity and viral clearance (32, 37–39). CD96 has been shown to be a marker for NK cell exhaustion in patients with hepatocellular carcinoma (HCC) and to limit antitumor functions of NK cells in mice (40, 41). Binding of TIGIT leads to decreased NK cell cytotoxicity and is suggested to ensure self-tolerance, as well as to promote liver regeneration (26, 32, 42). In this study, we performed a detailed characterization of CD56^bright^ NK cells derived from matched peripheral blood and liver tissues on the transcriptional level and by assessing protein expression profiles, and determined their functional activity. The herein described results provide a deeper understanding of the differences between peripheral blood and tissue NK cell subsets, and the impact of the liver environment on CD56^bright^ NK cells.

## 2 Materials and methods

### 2.1 Participants

Matched peripheral blood and tumor-free liver samples were obtained from patients at the Asklepios Hospital Barmbek (AKB) undergoing extended liver resection due to liver metastases following colorectal cancer, liver adenoma, cholangiocellular carcinoma or hemangioma (n=17), and at the University Medical Center Hamburg-Eppendorf (UKE) from patients undergoing liver transplantation (n=17) due to end-stage liver disease. Human control peripheral blood samples were obtained from 13 healthy individuals. Furthermore, residual amounts of anonymized peripheral blood samples (buffy coats) from 12 randomly-selected healthy blood donors recruited at the Institute for Transfusion Medicine at the UKE were used. All blood donors gave their general written consent to use their blood samples for scientific studies. The anonymized use of buffy coats complied with a vote by the ethics committee of the German Medical Association. Study protocols (PV4898, PV4081, and PV4780) were approved by the ethics committee of the medical association of Hamburg, and all study participants provided written informed consent. The demographics and clinical characteristics of study participants are summarized in **Tables 1**, **2**.

**Table 1:** Demographics and clinical characteristics of patients included in this study.

	Figure 1	Figure 2	Figure 3	Figure 4	Figure 5	All
Samples						
Liver	4	19	8	–	–	27*
Matched PBMC	4	19	0	–	–	19*
Hepatocyte organoids		–	–	–	7	7**
Demographics						
Sex (f/m)	2/2	7/12	4/4	–	4/3	15/19
Median age in years (Range)	57 (43-68)	57 (43-68)	56 (45-65)	–	57 (50-75)	57 (43-75)
Primary liver disease (LTX)						17
ALD	0	4	0	–	–	4
HCV	0	2^‡^	0	–	–	2^‡^
HCC	0	5^†^	4^†^	–	–	9^†^
PLD	0	0	1	–	–	1
PSC	0	0	1	–	–	1
Primary liver disease (AKB)						17*
Colorectal carcinoma	3	7	2	–	5	14*
Inflammatory adenoma	1	1	0	–	0	1*
Cholangiocellular carcinoma	0	0	0	–	1	1
Hemangioma	0	0	0	*-*	1	1
CMV serological status						
positive/negative/unknown	4/0/0	14/5/0	6/1/1	–	0/0/7	20/6/8

*Samples of 4 patients with eiter colorectal carcinoma metastasis or liver adenoma were used in two experiments.

**Hepatocyte organoids from 7 individuals were used for 15 experiments in figure 5.

^†^HCC was based on: 4x HCV, 1x HBV with HDV, 1x CC, 1x NASH and 2x ALD.

^‡^One patient suffered from HBV and HCV simultaneously.

AKB, Asklepios Hospital Barmbek liver resection cohort; ALD, alcoholic liver disease; CC, cryptogenic cirrhosis; CMV, cytomegalovirus; HBV, hepatitis B virus; HCC, hepatocellular carcinoma; HCV, hepatitis C virus; HDV, hepatitis D virus; LTX liver transplantation cohort; NASH, non-alcoholic steatohepatitis; PBMC, peripheral blood mononuclear cells; PLD, polycystic liver disease; PSC, primary sclerosing cholangitis.

**Table 2:** Demographics and characteristics of healthy donors included in this study.

*Healthy donors*	Figure 1	Figure 2	Figure 3	Figure 4	Figure 5	*All*
**Samples**						
PBMC	2	5	–	8	–	13*
**Demographics**						
Sex (f/m)	2/0	4/1	–	5/3	–	9/4
Median age in years (Range)	27.5 (26-29)	29 (26-41)	–	33 (26-43)	–	33 (26-43)
**CMV serological status**						
positive/negative/unknown	0/2/0	0/3/2	–	0/3/5	–	0/6/7

*Samples of 2 healthy donors were used in two experiments.

CMV, cytomegalovirus; PBMC, peripheral blood mononuclear cells.

### 2.2 Sample processing

Blood sample and liver tissue preparation were performed as previously described (43–45). Peripheral blood mononuclear cells (PBMCs) were obtained using density gradient centrifugation with Ficoll (Biochrom GmbH, Berlin, Germany). Tumor-free liver tissue was dissolved mechanically without enzymatic digestion for the extraction of hepatocytes and intrahepatic NK cells. For liver organoid generation and culture, liver tissue was mechanically and enzymatically digested and cultured as previously described (15, 46). In brief, the liver tissue was cut into small pieces and washed with DMEM (Gibco) supplemented with 10% fetal bovine serum (FBS) and 1% Penicillin/Streptomycin. The tissue pieces were then incubated at 37°C in EBSS (Gibco) with Collagenase D (Sigma-Aldrich) and DNase I (STEMCELL) until single cells were microscopically detectable. The cell solution was then filtered and washed. Single cells were seeded in 50µl droplets of BME2 (Basement Membrane Extract, Type 2, Pathclear) and organoid culture medium (46) and passaged 3 to 4 weeks after the digestion. Further passaging was performed approximately after 7 days of culture. Medium was changed every 2-3 days. PBMCs and liver-derived cells were cryopreserved, stored in liquid nitrogen and thawed for immediate experimental use.

### 2.3 Antibody staining and flow cytometry

After thawing, liver-derived cells and PBMCs were washed with phosphate buffered saline (PBS) and stained with surface antibodies and the live-dead markers Zombie NIR or Zombie Aqua (BioLegend) for 20 minutes in the dark at room temperature. All antibodies used in this study and corresponding dilutions are listed in **Supplementary Table S1**. After washing with PBS, cells were either fixed immediately with 4% paraformaldehyde or fixed and permeabilized using the BD Cytofix/Cytoperm Kit (BD Bioscience), as recommended by the manufacturer, and subsequently stained with intracellular antibodies. The samples were measured using a LSRFortessa™ flow cytometer (BD Bioscience) or a Cytek Aurora (Cytek Biosciences). FlowJo™ version 10.7.1 was used to analyze the data. Gating strategies are shown in the supplementary information (**Figures S1A, B**).

### 2.4 Single-cell mRNA analysis

Gene expression analyses and data processing were performed as previously described (23, 47, 48). To assess single-cell mRNA expression of selected genes, a Fluidigm platform consisting of C1, Juno and Biomark HD was used. Matched PBMCs and liver-derived cells from four liver resection patients and PBMCs of two healthy donors were sorted for live, CD3^−^CD14^−^CD19^−^CD56^bright^CD16^−^ NK cells (**Figure S1A**) with a BD FACSAria Fusion (BD Bioscience) into a C1 Single-Cell Preamp Integrated Fluidic Circuit (IFC), 5-10 µm (Fluidigm). Cell lysing, reverse transcription, and preamplification were performed using the C1, according to company protocol (PN 100-4904 K1). 52 primers were used to quantify gene expression and are summarized in the supporting data (**Supplementary Table S2**). Quantitative PCR was performed using the 96.96 Dynamic Array IFC for Gene Expression in the Biomark HD. All data analysis was performed with the Fluidigm Real-Time PCR Analysis software (Version 4.3.1). The Limit of Detection (LOD) was defined as a Ct value of 24 and subsequently all Ct values above 24 were set to the LOD. Gene expression levels are displayed as 2^(LOD-Ct)^, thus a value of 1 indicates a cell without detectable mRNA expression of the respective gene (49, 50). In the Fluidigm Real-Time PCR Software, all peak detection ranges of the melting curves were defined as the median temperature peak of all non-failed results of the corresponding primer pair +/-1.2°C. Other settings remained unchanged (Peak Sensitivity: 7; Peak Ratio Threshold: 0.8). The Ct value was set to the LOD for all reactions that were marked as failed under these settings.

### 2.5 Degranulation assay with liver-derived NK cells

Liver-derived cells were thawed in pre-warmed RPMI (Thermo Fisher Scientific) supplemented with 10% FBS (R10) (Biochrom GmbH) and 10 µg/ml DNase. Subsequently, after centrifugation and washing, cells were resuspended at a concentration of 2x10^6^ cells/mL in R10 supplemented with 1 ng/mL IL-15 and rested overnight at 37°C. Cells were washed with PBS and counted before being co-incubated with K562 cells at an effector to target cell (E:T) ratio of 10:1 for 5h at 37°C in the presence of an anti-CD107a-antibody (BV711) and Brefeldin A. Hereafter, cells were stained with surface antibodies and Zombie NIR, as well as stained intracellularly with an anti-TNF-α-antibody (PE-Cy7), as described above.

### 2.6 Co-culture of peripheral blood NK cells with Huh7 cells

NK cell isolation was performed as recommended by the manufacturer (EasySep Human NK cell Isolation Kit, Stemcell Technologies) with fresh PBMCs from healthy donors. Isolated NK cells were stimulated for 12h with 1 ng/mL IL-15 at 37°C. Afterwards, NK cells were either (co-)incubated with or without Huh7 cells at an E:T ratio of 2:1 in direct contact or in indirect contact using a transwell insert (Sarstedt AG & Co. KG) with pore sizes of 1 µm for 6, 12, 24 and 48h. Finally, cells were stained with antibodies for flow cytometric analysis, as described above. For blocking experiments, Huh7 cells were co-incubated with an anti-PVR blocking antibody (30µg/ml) for 15 min prior to co-culture.

### 2.7 Co-culture of peripheral blood NK cells and human primary hepatocyte organoids

Hepatocyte organoids were thawed, cultured in 50 µl droplets containing reduced growth factor BME2 and culture medium, as previously described (46). Growing hepatocyte organoids were passaged once before experimental use. Fresh PBMCs from buffy coats were enriched for NK cells using the EasySep™ Human NK Cell Enrichment Kit (Stemcell Technologies) following manufacturers protocols. NK cells were resuspended in RPMI (Thermo Fisher Scientific) supplemented with 10% FBS (Biochrom GmbH) (R10) and 1% Penicillin/Streptomycin and were added to fully-grown hepatocyte organoids. After 24 and 48 hours of co-incubation, cells present in the supernatant or the droplet, respectively, were collected separately, treated with TripLE™ Express Enzyme (Life Technologies GmbH) for organoid dissociation, stained and analyzed by flow cytometry. As controls, NK cells were cultured in R10 supplemented with 1% Penicillin/Streptomycin, but no hepatocyte organoids, and subsequently equally treated with TripLE™ Express Enzyme and stained.

### 2.8 Multiplex assay

Supernatants from hepatocyte organoids were collected 48h after the last medium change and stored at -80°C until further analysis. The multiplex assay (Bio-Rad) was performed on a Bio-Plex 200 System. Secretion of various chemokines by hepatocyte organoids (CXCL1, CXCL5, CXCL9, CXCL10, CXCL11, CXCL12, CXCL16, CX3CL1, CCL2, CCL5, CCL19, CCL20, and CCL21) was measured.

### 2.9 Statistical analysis

Statistical analysis was performed using GraphPad Prism 8 and 9 (GraphPad Software, Inc., La Jolla, CA) or R (R 4.0.3 GUI 1.73; R Studio, Version 1.3.1093, lme4, ggplot2, FactoMineR and factoextra packages). Principal component analysis was generated with R using the mRNA expression of 52 genes of 440 single CD56^bright^ ihNK cells or pbNK cells. Wilcoxon matched-pairs signed rank test was used to determine differences between paired samples with assumed non-normal distribution. To assess normal distribution within small sample sizes, the Shapiro-Wilk test was applied and subsequently, the paired t test was used. A mixed-effects model with random intercept, considering intra-sample correlations, was used to compare the single-cell mRNA expression data from different donors (or groups). To limit the detection of false positives, the p-values were adjusted by the Benjamini and Hochberg False Discovery Rate (FDR) method between each comparison groups (cutoff of 0.05). P values <0.05 were considered statistically significant. Statistical analysis for additional data presented in the figures are provided in the respective figure legends.

### 2.10 Visualization of t-distributed stochastic neighbor embedding (viSNE)

Flow cytometry data were visualized using viSNE (Cytobank, Santa Clara, CA). A tool for visualization of high-dimensional data based on the Barnes-Hut implementation of the t-distributed stochastic neighbor embedding algorithm was used (51). NK cells were equally sampled to randomly select 50 000 cells for analysis. The analysis was performed using the surface expression of CD56, CXCR6, CD69, TIGIT, DNAM-1 and CD96. The following settings were adjusted: 2000 iterations, 20 perplexity, theta 0.5.

## 3 Results

### 3.1 Intrahepatic and matched peripheral blood CD56^bright^ NK cells differ in the expression of genes related to liver-residency, migration, and regulatory functions

**Figure 1:**
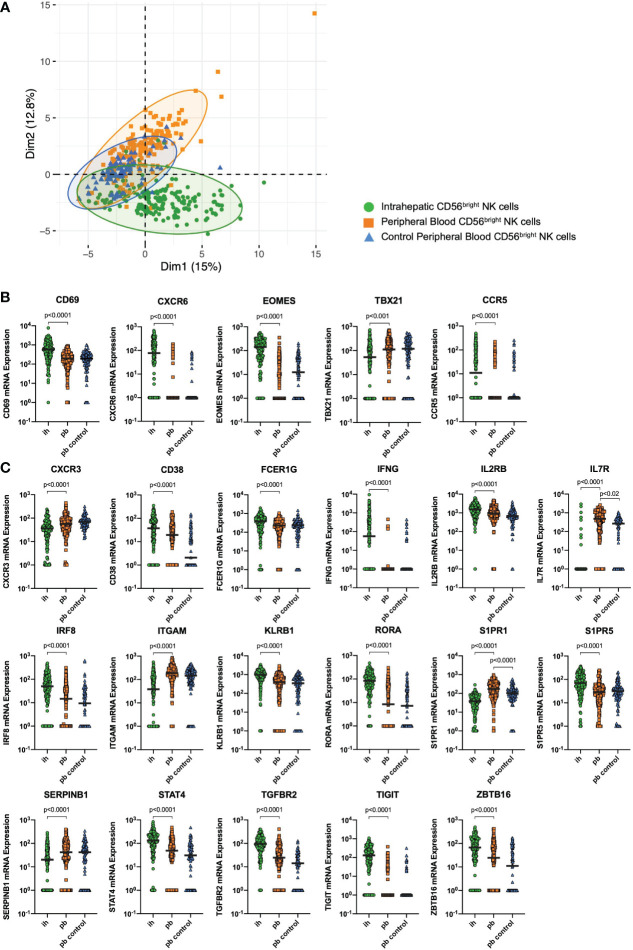
Single-cell mRNA expression. **(A)** Principal component analysis showing clustering of CD56^bright^ peripheral blood (pb)NK cells from healthy control individuals (blue triangles), CD56^bright^ pbNK cells from liver resection patients (orange squares) and CD56^bright^ intrahepatic (ih)NK cells from liver resection patients (green dots). **(B)** Quantitative analysis of CD56^bright^ ihNK cells and matched CD56^bright^ pbNK cells from liver resection patients (n=4), and CD56^bright^ pbNK cells from healthy control individuals (n=2) showing single-cell mRNA expression of liver-residency markers CD69, CXCR6, CCR5, Eomes and T-bet (*TBX21*). **(C)** Quantitative analysis of CD56^bright^ ihNK cells and matched CD56^bright^ pbNK cells from liver resection patients (n=4), and CD56^bright^ pbNK cells from healthy control individuals (n=2) showing single-cell mRNA expression of selected genes with highly significant differential gene expression comparing ih and pbNK cells. Green dots representing 178 ihNK cells, orange squares representing 173 pbNK cells and blue triangles representing 89 healthy control pbNK cells. A mixed-effects model with random intercept, considering intra-sample correlations, was used to compare the single-cell mRNA expression data from different donors (or groups). Black line indicates median.

Matched peripheral blood (pb) and intrahepatic (ih) NK cells from patients undergoing liver resection due to either liver metastases (n=3) or liver adenoma (n=1) were used for targeted single-cell mRNA expression analysis in this study. Furthermore, pbNK cells from healthy individuals (n=2) of the Hamburg Healthy Cohort were used as a control. We sorted CD56^bright^ ihNK cells and CD56^bright^ pbNK cells (**Figure S1A**) and investigated the single-cell mRNA expression of 52 representative genes on a total of 440 CD56^bright^ NK cells. All differentially expressed genes can be found in **Supplementary Table S3**. Principal component analysis (PCA) revealed divergence of the NK cells depending on their tissue origin. Thus, this unsupervised dimension reduction method showed two partly separating clusters, one for CD56^bright^ ihNK cells and another one for CD56^bright^ pbNK cells, the latter overlapping with the control group of CD56^bright^ pbNK cells (**Figure 1A**). The separation of the PCA-dimension-1 was mainly driven by 18 genes, including genes associated with NK cell activity (e.g. *CD38*, *NCR1, KLRB1*) and tissue-residency (e.g. *EOMES, TIGIT, CXCR6*) *(* 16, 22, 52–54). In conclusion, distinct single-cell gene expression discriminates CD56^bright^ pbNK cells and ihNK cells.

We next analyzed whether expression levels of mRNA of the known liver-residency markers CD69, CXCR6, CCR5, Eomes and T-bet differed between CD56^bright^ NK cells derived from livers and matched PBMCs (**Figure 1B**). Compared to CD56^bright^ pbNK cells, CD56^bright^ ihNK cells showed a significantly higher mRNA expression of *CD69* (p<0.0001), *CXCR6* (p<0.0001), *CCR5* (p<0.0001) and the transcription factor *EOMES* (p<0.0001), while the transcription factor T-bet (*TBX21*) showed significantly lower (p<0.0001) mRNA expression in CD56^bright^ ihNK cells. This distinct mRNA expression of liver-residency markers in CD56^bright^ ihNK cells is in line with previous results from studies characterizing the phenotype of CD56^bright^ ihNK cells (16, 18–20, 23). In addition to the mentioned differentially expressed known liver-residency markers, we identified 17 genes with a highly significant differential mRNA expression comparing single CD56^bright^ ihNK cells and pbNK cells (**Figure 1C**). CD56^bright^ ihNK cells showed significantly higher mRNA expression of *CD38*, *FCER1G, IFNG, IL2RB*, *IRF8*, *KLRB1, RORA, S1PR5, STAT4, TGFBR2, TIGIT* and *ZBTB16* (all p<0.0001). Most of these genes are primarily associated with regulatory immune functions, anti-inflammatory activity and tissue residency (55–60), while others are involved in pathways of NK cell proliferation (61). In contrast, CD56^bright^ pbNK cells expressed significantly higher levels of *CXCR3, IL7R, ITGAM, S1PR1* and *SERPINB1* (all p<0.0001), genes that are mainly associated with NK cell migration and maturation (62–67). Interestingly, CD56^bright^ ihNK cells that expressed high mRNA levels of *TIGIT* also encoded mostly (63%) for CXCR6 and CD69 mRNAs, two markers of liver residency, and also for the chemokine receptor CCR5, associated with tissue homing (see **Supplementary Figure S2**). Taken together, the analysis of gene expression on single NK cells identified significant differences between CD56^bright^ ihNK cells and CD56^bright^ pbNK cells, including the higher expression of genes involved in NK cell proliferation, regulatory functions, and tissue-residency in ihNK cells.

### 3.2 CD56^bright^ NK cells in liver and peripheral blood differentially express TIGIT, DNAM-1 and CD96

**Figure 2:**
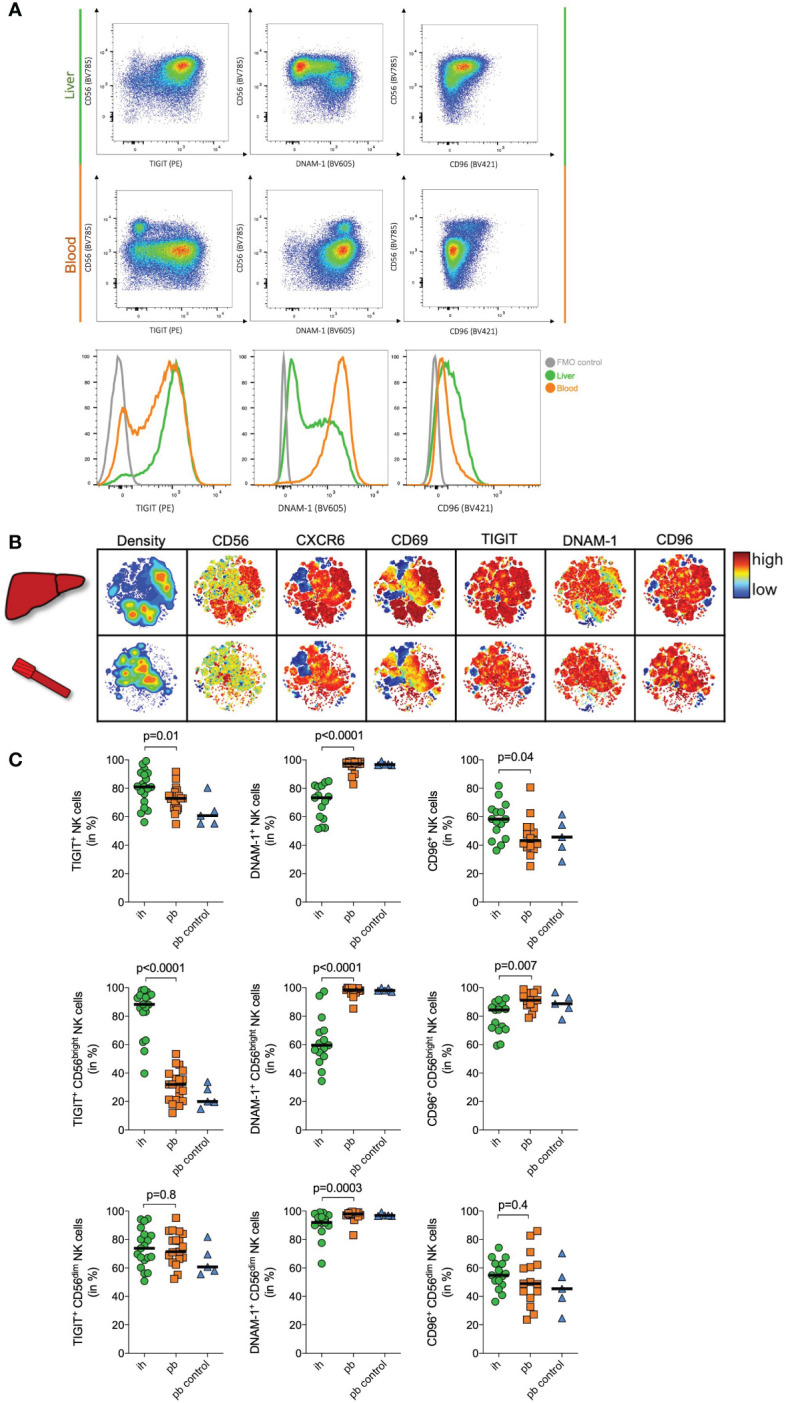
Immunophenotyping of NK cells. **(A)** Representative plots of flow cytometry data of one donor showing TIGIT, DNAM-1 and CD96 expression on peripheral blood (pb) and intrahepatic (ih) bulk NK cells. Histogram showing TIGIT (left), DNAM-1 (middle) and CD96 (right) expression on bulk NK cells in the liver (green), the blood (orange) and a FMO control (grey). **(B)** Representative viSNE plots showing density and the expression of CD56, CXCR6, CD69, TIGIT, DNAM-1 and CD96 on ih and pbNK cells from one patient undergoing liver transplantation. Color coding indicates the density and marker expression. viSNE analysis was performed using Cytobank. **(C)** Quantitative analysis of flow cytometry data of matched ih and pbNK cells from liver resection patients (n=15 for DNAM1- and CD96, n=19 for TIGIT), and control pbNK cells from healthy individuals (n=5) showing TIGIT, DNAM-1 and CD96 expression on bulk, CD56^dim^ and CD56^bright^ NK cells. Green dots representing ihNK cells, orange squares representing pbNK cells and blue triangles representing healthy control pbNK cells. Wilcoxon matched-pairs sign rank test was used to determine statistical differences between ih and pbNKcells in all scatter plots, Mann-Whitney test was used to determine statistical differences between pb and pb control NK cells in all scatter plots. Black line indicates median.

TIGIT is a co-inhibitory receptor that inhibits human NK cell cytotoxicity and has been suggested to be critical in murine liver regeneration (26, 42). The significantly elevated mRNA expression of TIGIT in CD56^bright^ ihNK cells prompted us to seek confirmation for this specific target on protein level by flow cytometry. We included TIGIT’s competing receptors DNAM-1 and CD96 in the analysis. All these receptors bind to the poliovirus receptor (PVR/CD155) and facilitate either immune activation or inhibition (32, 41). We analyzed TIGIT, DNAM-1 and CD96 surface protein expression on CD56^bright^ ihNK cells and matched CD56^bright^ pbNK cells from patients undergoing liver resection or liver transplantation (**Figure 2A**), as well as peripheral blood samples from healthy control individuals (**Figure 2C**). Frequencies of CD56^bright^ and CD56^dim^ NK cell subsets varied between ihNK cells and pbNK cells. CD56^bright^ NK cells were more frequent in ihNK cells compared to pbNK cells or healthy control NK cells (median frequency (%) of CD56^bright^ NK cells: 35%; 3,4%; 6,4%, respectively), while CD56^dim^ NK cells were less frequent in ihNK cells compared to pbNK cells or healthy control NK cells (median frequency (%) of CD56^dim^ NK cells: 40,1%; 88,7%; 82,5%, respectively).

A representative viSNE analysis revealed different clusters when comparing matched CD56^bright^ pbNK cells and ihNK cells from the same individual (**Figure 2B**). We identified clusters with high density and overlapping expression of CD56, liver-residency markers CD69, CXCR6, TIGIT and CD96. However, those clusters showed a distinctly lower expression of DNAM-1. When analyzing both subsets in detail, bulk ihNK cells showed higher TIGIT (p=0.01) and CD96 (p=0.04) expression (**Figure 2C**). In contrast, bulk pbNK cells showed a higher DNAM-1 expression (p<0.0001), which is consistent with a previous study demonstrating significantly higher DNAM-1 expression on pbNK cells (21). Regarding the CD56^dim^ NK cell subset, TIGIT (p=0.8) and CD96 (p=0.4) were equally expressed on pb and ihNK cells (**Figure 2C**). However, CD56^dim^ pbNK cells exhibited a significantly higher expression of DNAM-1 (p=0.0003). Differences in surface protein expression between pb and ihNK cells were most pronounced in the CD56^bright^ NK cell compartment (**Figure 2C**). In line with the corresponding mRNA data, we observed a significantly stronger surface protein expression of the inhibitory receptor TIGIT on CD56^bright^ ihNK cells than in CD56^bright^ pbNK cells (p<0.0001). In contrast, the CD56^bright^ pbNK cells showed significantly higher expression of DNAM-1 (p<0.0001) and CD96 (p=0.007) compared to their intrahepatic counterpart. Taken together, ihNK cells and especially the subset of CD56^bright^ ihNK cells showed higher levels of TIGIT, whereas pbNK cells distinctly exhibited higher expressions of DNAM-1.

### 3.3 Intrahepatic TIGIT^+^ CD56^bright^ NK cells exhibit diminished degranulation and TNF-α production

**Figure 3:**
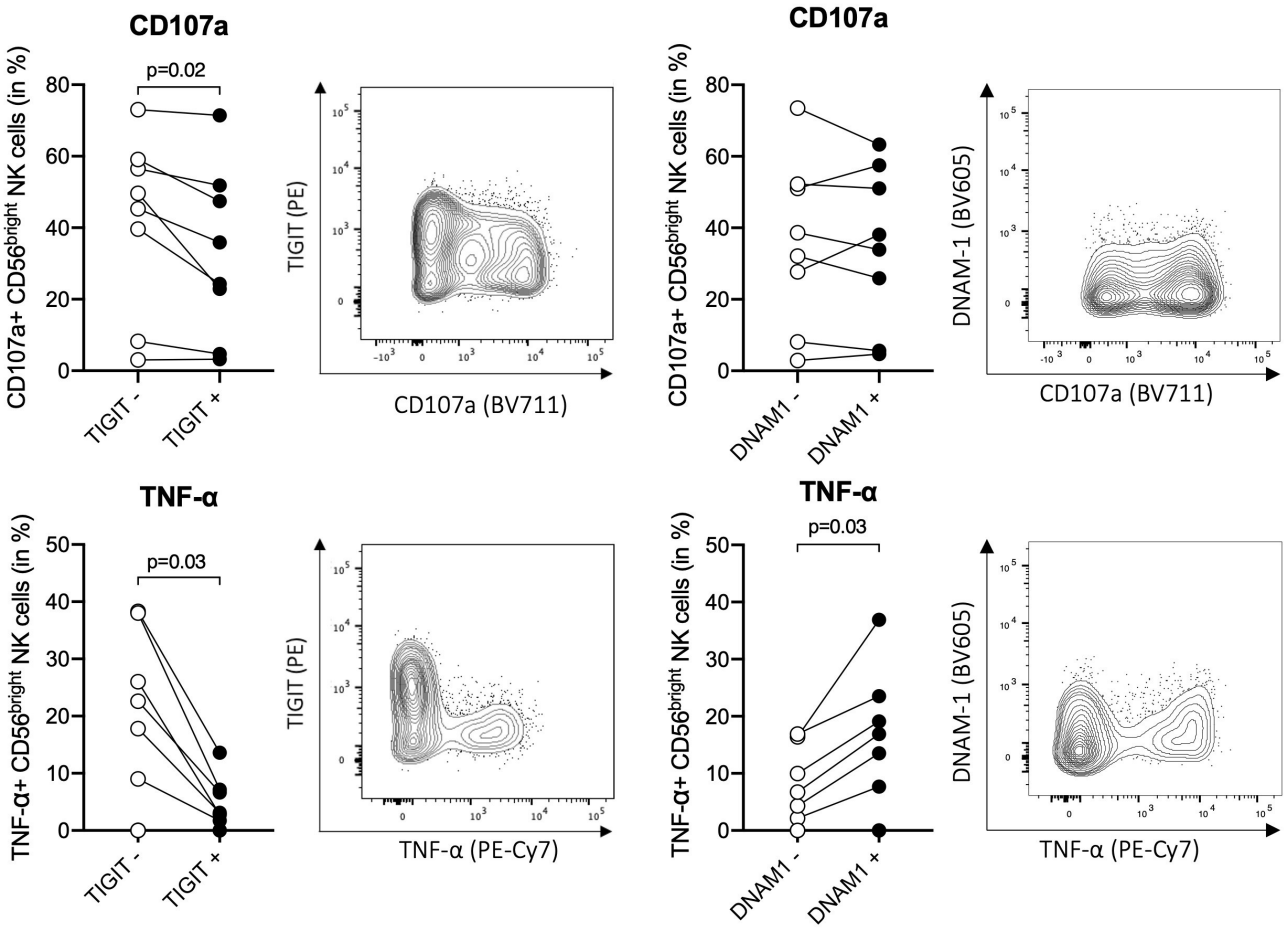
Functional activity of TIGIT^+/–^ and DNAM-1^+/–^ cells. Functional responses of TIGIT^+/–^ and DNAM-1^+/–^ CD56^bright^ ihNK cells, following co-incubation of liver-derived cells with K562 (E:T ratio of 10:1) for 5h at 37°C. Representative flow plots showing distribution of CD107a (top) and TNF-α (bottom) expression on CD56^bright^ ihNK cells depending on their TIGIT or DNAM-1 expression. Graphs on top showing percentage of CD107a^+^ TIGIT^+/–^ or DNAM-1^+/–^CD56^bright^ ihNK cells and graphs below showing percentage of TNF-α^+^ TIGIT^+/–^ or DNAM-1^+/–^CD56^bright^ ihNK cells. Wilcoxon matched-pairs sign rank test was used to determine statistical differences. Each dot represents one donor (n=8).

Previous data in mice and humans have suggested that TIGIT^+^ NK cells are functionally exhausted (68, 69), but the role of TIGIT in human ihNK cells is not well understood. To assess functional differences between TIGIT^+^ and TIGIT^–^ CD56^bright^ ihNK cells, we next determined tumor necrosis factor alpha (TNF-α) production and degranulation (CD107a expression) after stimulation of intrahepatic cells from patients undergoing liver transplantation (n=6) or liver metastases resection (n=2) with K562 target cells (**Figure 3**). After stimulation, we observed significantly less degranulation of TIGIT^+^ CD56^bright^ ihNK cells compared to TIGIT^–^ CD56^bright^ in NK cells (p=0.02). Moreover, the TIGIT^+^ CD56^bright^ ihNK cells also exhibited a significantly diminished TNF-α response compared to TIGIT^–^ CD56^bright^ ihNK cells (p=0.03). Overall, TIGIT^+^ CD56^bright^ ihNK cells were significantly less responsive to stimulation than TIGIT^–^ CD56^bright^ ihNK cells, in line with previous findings describing reduced functionality of TIGIT^+^ NK cells (68, 69).

### 3.4 Co-culture of peripheral blood NK cells with human hepatoma cells and hepatocyte organoids induced NK cell migration and an intrahepatic TIGIT^+^ NK cell phenotype

**Figure 4:**
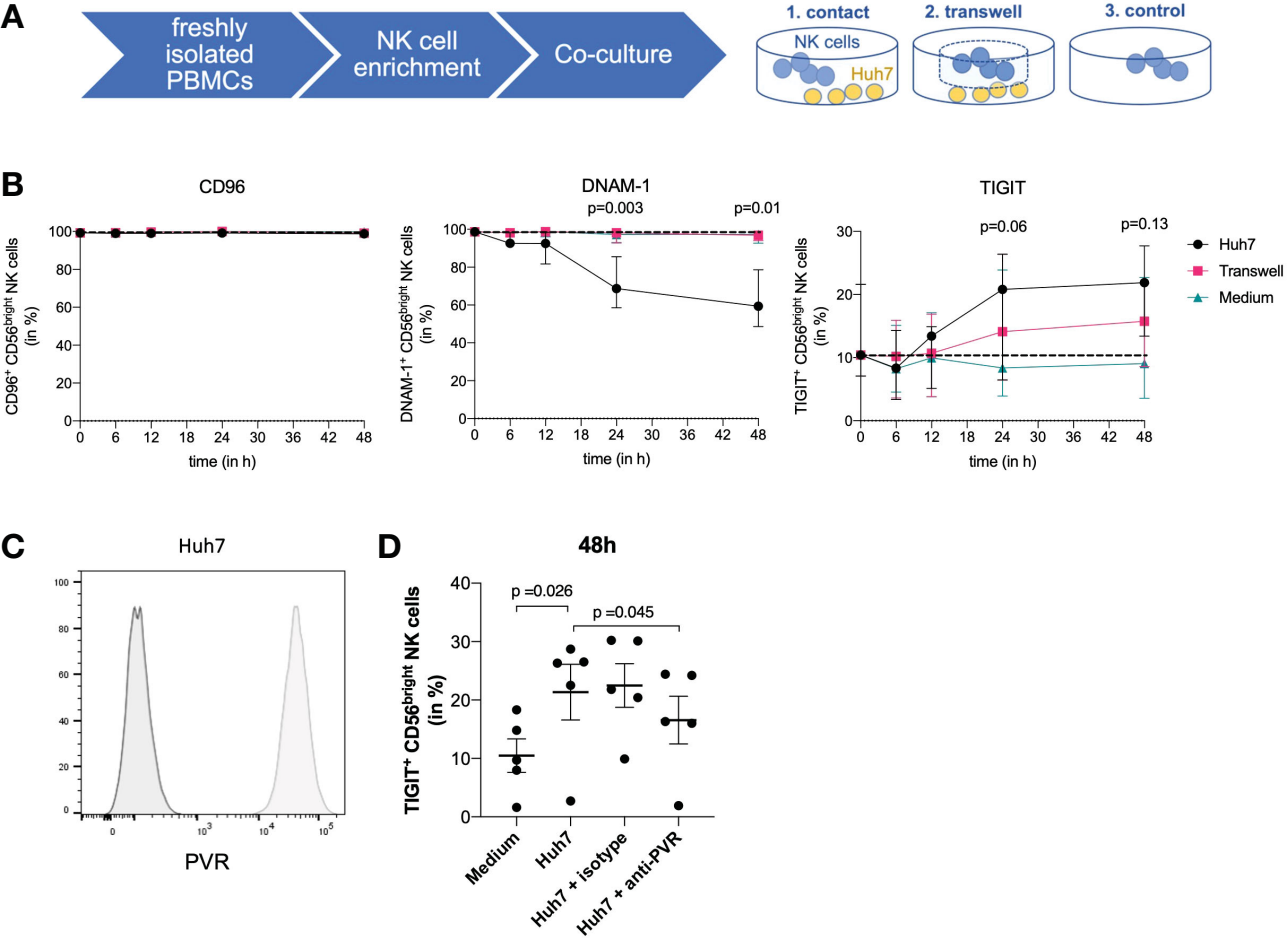
Co-culture of NK cells and Huh7 cells. **(A)** Co-culture experiments using isolated NK cells from peripheral blood from healthy control individuals and Huh7 cells. NK cells were either cultured in direct contact with Huh7 cells, in a transwell insert with the size of 1 µm to ensure indirect contact with Huh7 cells or with medium only. **(B)** Graphs are displaying quantitative analysis of CD96, DNAM-1 and TIGIT expression on CD56^bright^ NK cells after 6, 12, 24 and 48 hours. At the timepoints 6, 12, and 24 hours a total of 5 healthy control individuals was used for comparison. At 48 hours, a total of 4 out of the 5 mentioned healthy control individuals were assessed for CD96, DNAM-1 and TIGIT. Black dots representing direct contact, pink squares the transwell insert and blue triangles medium only. Paired t-test was used to determine the displayed statistical differences between direct contact of NK cells and medium control. Each dot represents the median of the individuals; error bars represent the data range. **(C)** Representative histograms comparing PVR staining (light grey) and unstained control (dark grey) demonstrating PVR expression on Huh7 cells. **(D)** Graph displays the percentage of TIGIT^+^ NK cells in different conditions: NK cells only, NK cells in direct contact with Huh7 cells, isotype control and NK cells in direct contact with Huh7 cells including blocking of PVR with an anti-PVR-antibody. Data are shown for 5 paired experiments using NK cells derived from 3 different donors. Statistical analysis for significance was performed using a two-tailed paired T test.

The above data demonstrate that CD56^bright^ ihNK cells express higher levels of TIGIT on both mRNA and protein levels, and that TIGIT^+^ NK cells show reduced functionality. We next determined whether the liver environment itself promoted an increased immunotolerance of ihNK cells, as the ligand for TIGIT, PVR/CD155, is expressed by hepatocytes (42). For this purpose, we co-cultured isolated pbNK cells from healthy individuals with the human hepatoma cell line Huh7, which stably expresses PVR (**Figure 4C**). To be able to differentiate between effects of direct receptor binding or soluble stimulation, NK cells were cultured either in direct contact with Huh7 cells or separated *via* a transwell insert (**Figure 4A**). Subsequently, the expression of TIGIT, DNAM-1 and CD96 was assessed (**Figure 4B**). Regardless of the condition, we did not observe any differences in CD96 expression up to 48 hours of co-culture. DNAM-1 expression in CD56^bright^ NK cells started to decline after 6 hours and continued to decrease up to 48 hours during co-culture, but only when in direct contact with the Huh7 cells (p=0.003). After 48 hours of co-incubation, DNAM-1 expression decreased significantly by almost 40% (p=0.01). However, no effect on the modulation of DNAM-1 expression was observed in the transwell experiments or in the absence of Huh7 cells, suggesting that direct cell-to-cell contact between NK cells and hepatoma cells was required to induce DNAM-1 downregulation. In contrast, TIGIT expression started to increase within 12 hours upon direct contact with Huh7 cells. This increase was even more pronounced after 24 hours of co-culture of CD56^bright^ NK cells in direct contact with Huh7 cells. Again, no effect on the modulation of TIGIT expression was detectable in CD56^bright^ NK cells cultured without Huh7 cells, and only minor changes in TIGIT expression were detected in transwell experiments. To start to elucidate the contribution of different factors to the observed TIGIT upregulation on NK cells, we compared the receptor expression of TIGIT on NK cells after direct co-incubation with Huh7 cells alone or in the presence of an anti-PVR blocking antibody (**Figure 4D**). As observed before, direct co-culture with Huh7 cells resulted in a significant upregulation of TIGIT on NK cells, which was significantly reduced, but not fully suppressed, by addition of the PVR-blocking antibody. Taken together, these data show that CD56^bright^ pbNK cells altered their phenotypes towards CD56^bright^ ihNK cells when cultured together with PVR-expressing human hepatoma cells, by downregulating activating and simultaneously upregulating inhibitory NK cell receptors that bind to PVR. These data suggest a direct impact of the liver environment on the phenotype and functional activity of ihNK cells.

**Figure 5:**
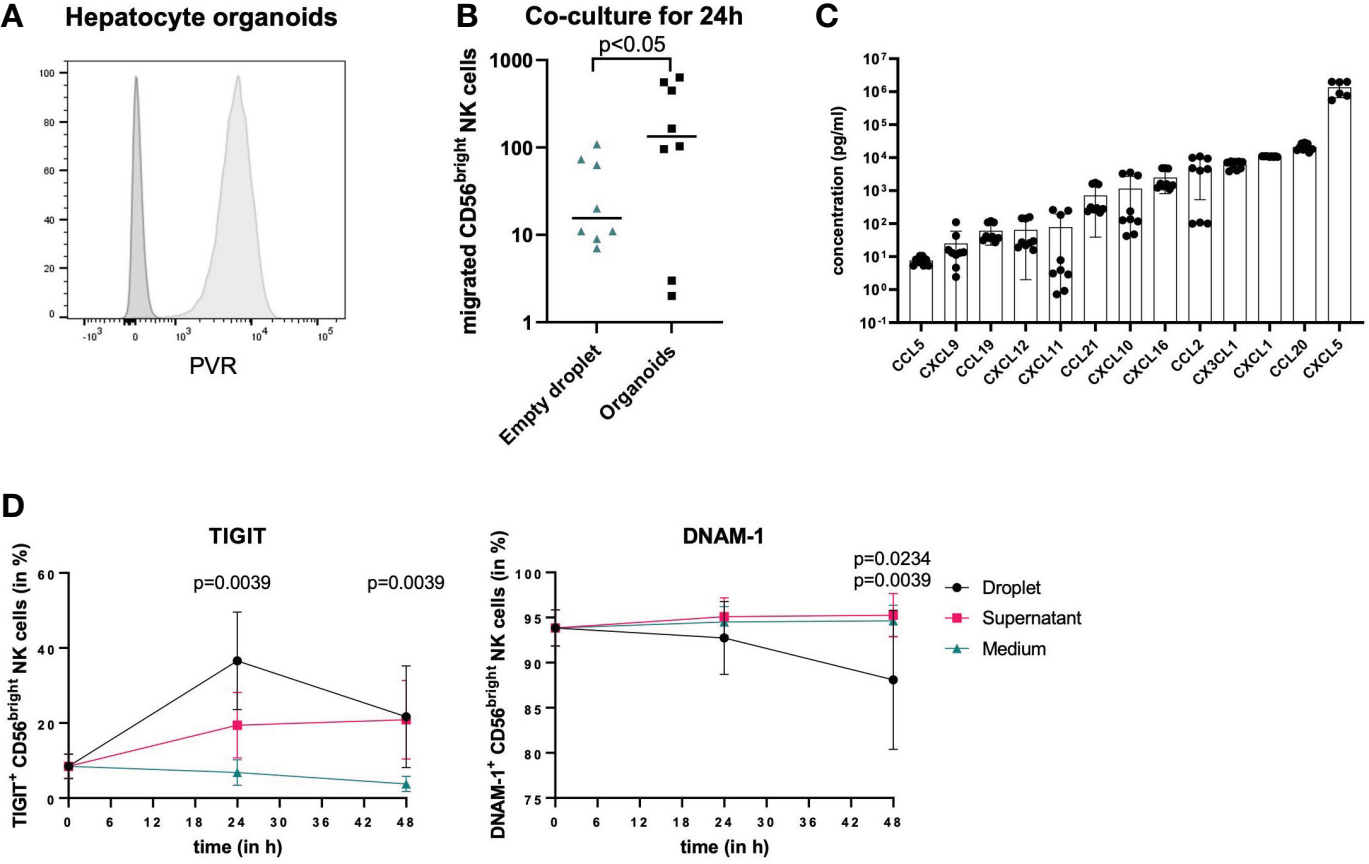
Co-culture of NK cells and hepatocyte organoids. **(A)** Representative histograms comparing PVR staining (light grey) and unstained control (dark grey) of hepatocyte organoids demonstrating PVR expression on hepatocytes. **(B)** NK cells isolated from buffy coats were co-cultured with empty BME2 droplets or hepatocyte organoids in BME2 droplets for up to 48h. Graph displaying total cell count of CD56^bright^ NK cells that migrated into BME2 droplets at 24 hours of co-culture with empty droplets (blue triangles) or hepatocyte organoids (black dots). Wilcoxon matched-pairs sign rank test was used for statistical analysis. Black line indicates median. (n=3, triplicates) **(C)** Chemokine concentrations in hepatocyte organoid culture supernatants are shown in ascending order on a logarithmic scale. Bars represent mean values of three experiments in triplicates for each chemokine and error bars show standard deviations (n=3, triplicates). **(D)** Migrated CD56^bright^ NK cells (black dots) were phenotypically compared to non-migrated cells (pink squares) and to separately cultured NK cells that did not have any contact to hepatocyte organoids (blue rectangles). Percentage surface expression of TIGIT and DNAM-1 is shown in CD56^bright^ NK cells after 24 hours and 48 hours. For TIGIT, statistical differences represent comparison of migrated NK cells as well as non-migrated NK cells vs. NK cells in medium and for DNAM-1 statistical differences represent migrated NK cells vs. NK cells in medium (top value) or non-migrated cells (lower value). Wilcoxon matched-pairs sign rank test was used for statistical analysis. Each dot represents the median of nine experiments; error bars represent the data range.

To further determine whether the observed changes in pbNK cells in the presence of hepatoma cells were also observed in response to human primary hepatocytes, we co-cultured hepatocyte organoids, which stably express PVR (**Figure 5A**) and were derived from human liver tissue and were growing within BME2 droplets with pbNK cells. pbNK cells migrate towards hepatocyte organoids, as the number of NK cells entering hepatocyte organoids and the surrounding BME2 droplets was significantly higher in wells with hepatocyte organoids compared to wells only containing empty BME2 droplets after 24 hours (**Figure 5B**). To assess which chemokines attracting NK cells are secreted by hepatocyte organoids, chemokine levels in the cell-culture supernatant from hepatocyte organoids were analyzed (**Figure 5C**). Chemokine levels were detected at different concentrations ranging from mean concentrations below 100 pg/ml (CCL5, CXCL9, CCL19, CXCL12, CXCL11) to concentrations above 700 pg/ml (CCL21, CXCL10, CXCL16, CCL2, CX3CL1, CXCL1, CCL20, CXCL5) (**Figure 5C**). The results from these co-culture studies demonstrate that hepatocyte organoids secrete chemokines that attract NK cells.

To identify phenotypical changes in CD56^bright^ pbNK cells following migration into hepatocyte organoids, we subsequently analyzed TIGIT and DNAM-1 surface expression of CD56^bright^ NK cells upon co-culture with hepatocyte organoids compared to CD56^bright^ NK cells in medium only (controls), and furthermore differentiated those NK cells that entered hepatocyte organoids within BME2 droplets from those NK cells that remained outside of BME2 droplets. Surface expression of TIGIT and DNAM-1 on NK cells was analyzed after 24 and 48 hours of co-culture (**Figure 5D**). Both, CD56^bright^ NK cells that entered BME2 droplets and those that did not showed an increased TIGIT expression after co-culture with hepatocyte organoids compared to control conditions. However, the percentage of TIGIT^+^ NK cells that migrated into hepatocyte organoids was already significantly higher after 24 hours compared to control conditions without organoids, while the frequency of TIGIT^+^ NK cells outside of the BME2 droplet (supernatant) increased more slowly (**Figure 5D**). Furthermore, DNAM-1 expression on NK cells continuously decreased only on those NK cells that entered the hepatocyte organoids, reaching significantly lower levels after 48 hours of co-incubation, while no decrease in percentages of DNAM-1 expressing NK cells was observed under the other conditions (**Figure 5D**). These data suggest that TIGIT and DNAM-1 expression on NK cells is preferentially modulated by direct cell-to-cell-contact and that primary human hepatocyte organoids can be used as an *in vitro* model to study migration of immune cells and their interactions with tissue cells.

## 4 Discussion

The liver plays a central role in mediating both local and systemic tolerance to self and foreign antigens, and this immune-regulatory capacity has been attributed to specialized liver-resident cells. NK cells make up 40% of total lymphocytes in human livers, and increasing evidence indicates that NK cells play an important role in the regulation of tissue-specific immunity (6). Liver-resident NK cells are mainly CD56^bright^ NK cells that express a distinctive profile of surface receptors and transcription factors (15, 16, 18–20). Unraveling NK cell functions that contribute to tissue homeostasis in the liver is critical to acquire a better understanding of hepatic immune regulation. In this study, we report transcriptional, functional, and phenotypical differences between CD56^bright^ ihNK cells and CD56^bright^ pbNK cells. CD56^bright^ ihNK cells exhibited significant upregulation of genes associated with anti-inflammatory activity and cell proliferation, whereas CD56^bright^ pbNK cells expressed higher levels of genes involved in NK cell migration and development. These data confirm that CD56^bright^ ihNK cells and CD56^bright^ pbNK cells represent two distinct NK cell populations and demonstrate that CD56^bright^ ihNK cells acquire phenotypical and functional characteristics associated with reduced responsiveness, potentially contributing to the tolerogenic environment of the liver.

Data on distinct gene expression profiles of CD56^bright^ ihNK cells presented here are in line with data from gene expression analyses of liver perfusates and peripheral blood (20), as well as with findings of single-cell RNAseq analyses revealing distinct liver NK cell populations (70–73). Our PCA analyses reflected clear divergence of NK cells depending on their tissue origin, while small differences in gene expression of CD56^bright^ pbNK cells between individuals with liver diseases and healthy controls, which might have been impacted by additional variables such as sex, age, or disease status. In ihNK cells, we observed an upregulation of the co-inhibitory receptor TIGIT on CD56^bright^ NK cell populations that also expressed the known liver-residency markers CD69 and CXCR6. The differences in the expression of TIGIT, DNAM-1 and CD96 surface receptors, which all interact with PVR, were most striking when comparing CD56^bright^ ihNK cell to pbNK cell subsets. Consistent with previous findings (40, 68, 74), we observed a functional impairment of TIGIT^+^ CD56^bright^ ihNK cells after co-incubation with K562 target cells. Importantly, co-culture with PVR-expressing human hepatoma cells or primary hepatocyte organoids enhanced TIGIT expression and reduced DNAM-1 expression on CD56^bright^ pbNK cells, suggesting a potential influence of the liver environment on the phenotype and function of ihNK cells.

Given the significant differences in mRNA expression profiles between CD56^bright^ ihNK cells and CD56^bright^ pbNK cells, our phenotypical and functional studies focused on the co-inhibitory receptor TIGIT and its competing receptors DNAM-1 and CD96. Since the first identification of TIGIT (26–28), multiple studies have shown that TIGIT can promote T cell suppression and that it is also involved in impairing NK cell effector functions (27, 68). A recent study of Doyle and colleagues correlated better liver functions in HCV patients with a greater abundance of CD56^bright^ ihNK cells (75). Additionally, the study demonstrated that portal vein blood contained high plasma levels of IL-10. Interestingly, TIGIT can promote IL-10 production of dendritic cells following engagement of PVR, resulting in a reduction of T cell functions (27, 75). Furthermore, Bi et al. described TIGITs role as a safeguard molecule in liver regeneration by negatively regulating NK-hepatocyte crosstalk in mice (42). In our study, ihNK cells and in particular the subset of CD56^bright^ ihNK cells exhibited higher levels of TIGIT, whereas pbNK cells expressed higher levels of DNAM-1. These differences in receptor profiles resulted in functional differences, since TIGIT^+^ NK cells were significantly less responsive to stimulation than DNAM-1^+^ NK cells. While this reduced responsiveness of TIGIT^+^ CD56^bright^ ihNK cells could contribute to the tolerogenic immune environment of the liver, it might also represent a disadvantage in the context of infections and cancers, resulting in a “sanctuary compartment” within the liver, as observed in the context of chronic infections with hepatotropic viruses. Furthermore, several studies have provided increasing evidence that high TIGIT expression is observed on tumor infiltrating NK cells and is associated with their functional exhaustion (40, 68), and that TIGIT blocking can boost anti-tumor immunity (41, 68).

To better understand the mechanisms underlying TIGIT upregulation and DNAM-1 downregulation on CD56^bright^ ihNK cells, we co-incubated pbNK cells with hepatoma cells or hepatocyte organoids in direct cell-to-cell contact or in a transwell setup, and compared the differential expression levels of TIGIT and DNAM-1 on CD56^bright^ pbNK cells. While we observed a small increase in TIGIT expression on CD56^bright^ pbNK cells co-cultured with hepatoma cells in the transwell condition, CD56^bright^ pbNK cells upregulated TIGIT expression more strongly following direct contact with human hepatoma cells, and this upregulation was significantly reduced, but not fully suppressed, in the presence of an antibody blocking PVR. During co-cultivation with hepatocyte organoids, CD56^bright^ pbNK cells migrated towards the hepatocyte organoids, which released several chemokines that act as chemoattractants to NK cells. The release of CXCL1, CX3CL1, CXCL10 and CCL21 in the cell-culture supernatant of hepatocyte organoids might have contributed to NK cell migration, as these chemokines are established NK cell attractants (76, 77). Particularly, CXCL10 might have attracted CD56^bright^ pbNK cells, as the receptor CXCR3 is involved in NK cell migration and homing and is especially expressed on CD56^bright^ pbNK cells (77). Furthermore, CD56^bright^ NK cells express CCR7 that interacts with CCL21, which therefore might also be involved in the observed migration (76, 77). Additionally, CX3CL1 can contribute to NK cell attraction by serving as a ligand for CX3CR1, which regulates NK cell migration and is expressed, at least at low levels, on CD56^bright^ NK cells (78).

Similar to CD56^bright^ pbNK cells that were co-cultured with hepatoma cells, CD56^bright^ pbNK cells that migrated towards primary hepatocyte organoids preferentially upregulated TIGIT expression. These data indicate that direct cellular interactions with hepatocytes expressing PVR might contribute to the induction of TIGIT expression on NK cells. Furthermore, co-incubation of peripheral blood-derived CD56^bright^ NK cells with Huh7 cells and hepatocyte organoids resulted in a pronounced downregulation of DNAM-1, in line with previous studies showing that physical binding to PVR can downregulate DNAM-1 on NK cells (69, 79). Taken together, our data using primary human NK cells in co-culture with primary hepatocyte organoids show that these 3D organoid systems can be used to study interactions between human immune and tissue cells *in vitro*, and suggest that TIGIT upregulation and DNAM-1 downregulation are parallel events promoted by direct contact of NK cells with PVR expressed on hepatic cells, representing an adaptation of circulating NK cells that home to the tolerogenic environment of the liver.

## Data availability statement

Storage of data is performed by the Leibniz Institute of Virology on an internal server. Raw data will be made available upon request and can be shared after confirming that data will be used within the scope of the originally provided informed consent. Requests to access these datasets should be directed to the corresponding author.

## Ethics statement

Study protocols (PV4898, PV4081, and PV4780) were approved by the ethics committee of the medical association of Hamburg. The patients/participants provided their written informed consent to participate in this study.

## Author contributions

AZ, LM, PF, MB, SL and MA designed the study. AZ, LM, PF, KH, GM, LH, GR, SML, SB, BP, TP, AN, and SL performed experiments. AZ, LM, PF, and KH analyzed the data. SH gave critical intellectual input. JH and SH helped design and perform mRNA analysis. MR performed statistical analysis of mRNA data and LR gave statistical guidance. AZ wrote the manuscript with the support of PF, LM, MB, SL, and MA. KO, SP, and LF provided patient samples. All authors critically reviewed the manuscript. All authors contributed to the article and approved the submitted version.
