# Supplementary material for: The co-inhibitory receptor TIGIT regulates NK cell function and is upregulated in human intrahepatic CD56bright NK cells
Source: Front Immunol. 2023 Feb 9;14:1117320. doi: 10.3389/fimmu.2023.1117320 (PMC9948018; doi:10.3389/fimmu.2023.1117320)
Supplement: Supplementary file 1 [file DataSheet_1.pdf]

Supplementary Material

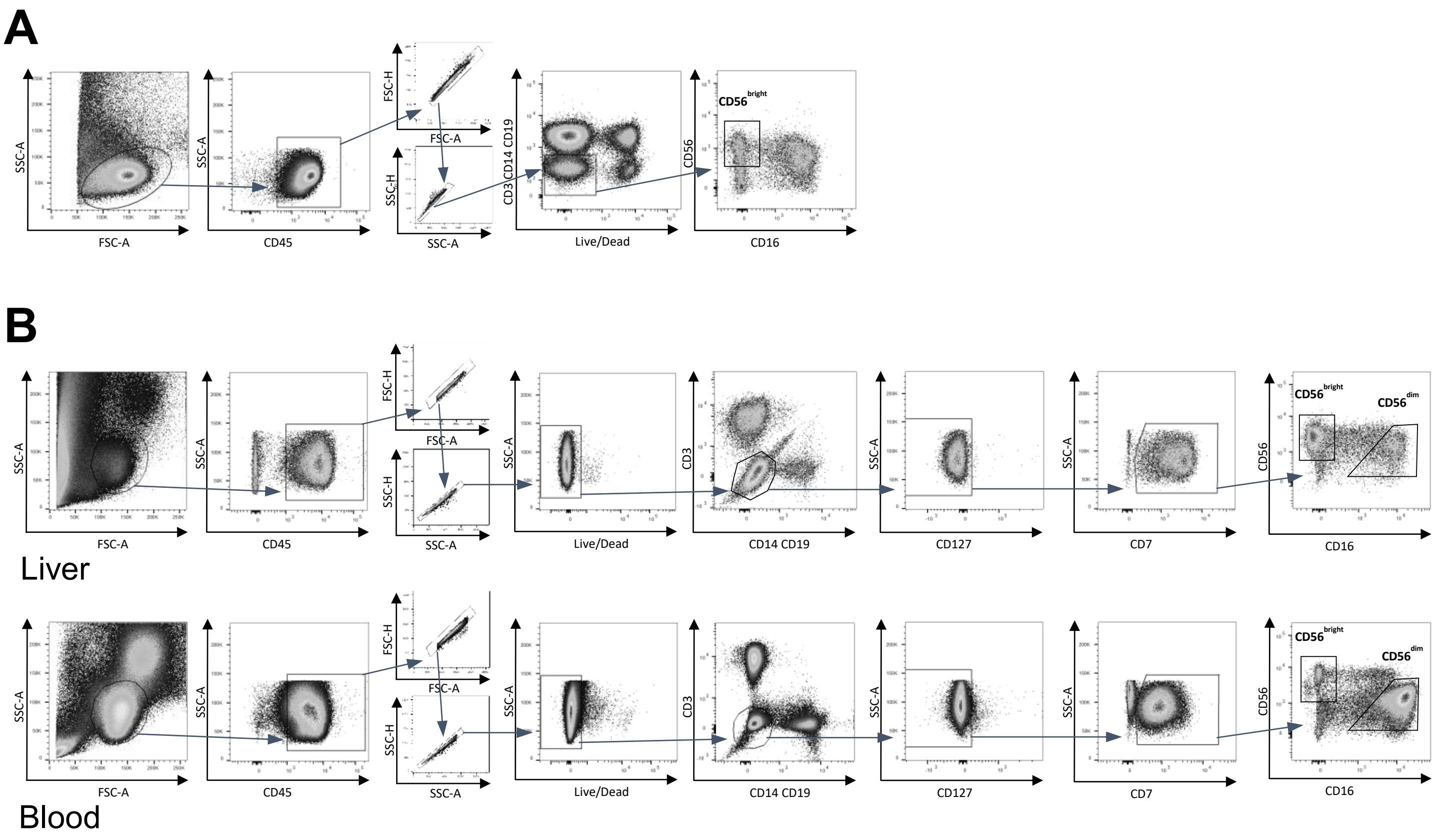

Supplementary Figure S1: Gating strategy for the identification of NK cells for single-cell mRNA analysis (A) and for phenotypical analysis (B).

Representative plots for the identification of CD56^{bright} NK cells from liver samples (A+B) and PBMCs (B). (A) NK cells for single-cell mRNA analysis were defined as CD45^{+}CD3^{-}CD14^{-}CD19^{-}CD16^{-}CD56^{bright} NK cells (B) NK cells for further phenotypical analysis were defined as CD3^{-}CD14^{-}CD19^{-}CD127^{-}CD7^{+}CD16^{-}CD56^{bright} NK cells or CD3^{-}CD14^{-}CD19^{-}CD127^{-}CD7^{+}CD16^{+} CD56^{dim} NK cells.

Initial gating on FSC-Area and SSC-Area. Lymphocytes were identified using CD45. Exclusion of doublets (FSC Width and SSC Width). Zombie NIR was used as a Live/Dead marker.

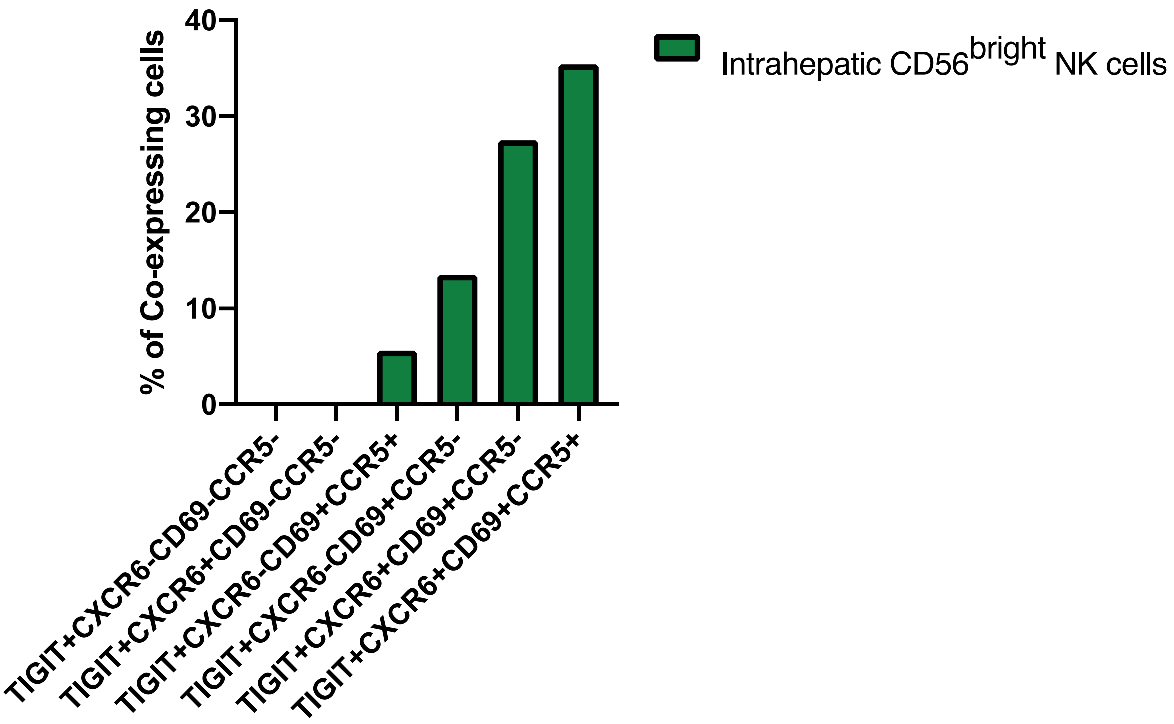

Supplementary Figure S2: Co-expression analysis of *TIGIT* and markers for tissue-residency and/or tissue-homing in intrahepatic CD56^{bright} NK cells.

Graph displaying percentage of mRNA co-expression of *TIGIT* and markers for liver-residency and/or tissue-homing (*CXCR6, CD69, CCR5*) in intrahepatic CD56^{bright} NK cells.

Supplementary Table S1: Antibodies used for flow cytometry.

Antibody/Reagent (Dilution)	*Fluorochrome*	*Clone*	*Supplier*	*Cat#*
**anti-CD3 (1:100)**	BV510	UCHT1	BioLegend, San Diego, CA, USA	300448
**anti-CD3 (1:100)**	FITC	UCHT1	BioLegend, San Diego, CA, USA	300406
**anti-CD3 (1:100)**	BUV395	UCHT1	BD Bioscience, San Jose, CA, USA	563546
**anti-CD7 (1:100)**	BUV395	M-T701	BD Bioscience, San Jose, CA, USA	563845
**anti-CD14 (1:100)**	AF700	M5E2	BioLegend, San Diego, CA, USA	325614
**anti-CD14 (1:100)**	BV510	M5E2	BioLegend, San Diego, CA, USA	301842
**anti-CD16 (1:100)**	APC-Cy7	3G8	BioLegend, San Diego, CA, USA	302018
**anti-CD16 (1:100)**	BV785	3G8	BioLegend, San Diego, CA, USA	302046
**anti-CD16 (1:100)**	BUV737	3G8	BD Bioscience, San Jose, CA, USA	564434
**anti-CD16 (1:100)**	PE/Cy7	3G8	BioLegend, San Diego, CA, USA	302016
**anti-CD19 (1:100)**	AF700	HIB19	BioLegend, San Diego, CA, USA	302226
**anti-CD19 (1:100)**	BV510	HIB19	BioLegend, San Diego, CA, USA	302242
**anti-CD45 (1:100)**	BUV395	HI30	BD Bioscience, San Jose, CA, USA	563792
**anti-CD45 (1:100)**	AF700	2D1	BioLegend, San Diego, CA, USA	368514
**anti-CD45 (1:100)**	BV711	HI30	BD Bioscience, San Jose, CA, USA	564357
**anti-CD56 (1:100)**	BUV395	NCAM16.2	BD Bioscience, San Jose, CA, USA	563554
**anti-CD56 (1:50)**	BUV737	NCAM16.2	BD Bioscience, San Jose, CA, USA	564447
**anti-CD56 (1:50)**	BV786	NCAM16.2	BD Bioscience, San Jose, CA, USA	564058
**anti-CD69 (1:100)**	BV421	FN50	BioLegend, San Diego, CA, USA	310930
**anti-CD69 (1:100)**	BV605	FN50	BioLegend, San Diego, CA, USA	310938
**anti-CD69 (1:100)**	APC	FN50	BioLegend, San Diego, CA, USA	310910
**anti-CD96 (1:50)**	BV421	NK92.39	BioLegend, San Diego, CA, USA	338418
**anti-CXCR6 (1:100)**	PE-Cy7	K041E5	BioLegend, San Diego, CA, USA	356012
**anti-CXCR6 (1:100)**	PerCP/Cy5.5	K041E5	BioLegend, San Diego, CA, USA	356010
**anti-TNFα (1:100)**	PE/Cy7	MAb11	BioLegend, San Diego, CA, USA	502930
**anti-CD107a (1:50)**	BV711	H4A3	BioLegend, San Diego, CA, USA	328640
**anti-CD127 (1:100)**	AF700	A019D5	BioLegend, San Diego, CA, USA	351344
**anti-CD226 (DNAM-1) (1:50)**	BV605	11A8	BioLegend, San Diego, CA, USA	338302
**Anti-CYP3A4 (1:50)**	PE		Biorbyt Ltd, Cambridge, UK	Orb124579
**Anti-PVR (1:50)**	PE	SKII.4	BioLegend, San Diego, CA, USA	337610
**Anti-PVR (1:50)**	PE-Cy7	SKII.4	BioLegend, San Diego, CA, USA	337614
**anti-TIGIT (1:50)**	PE	A15153G	BioLegend, San Diego, CA, USA	372704
**Mouse IgG1, κ isotype control (1:200)**	PE	MOPC-21	BioLegend, San Diego, CA, USA	400112
**Mouse IgG1, κ isotype control (1:200)**	PE-Cy7	MOPC-21	BioLegend, San Diego, CA, USA	400125
**Zombie Aqua Fixable Viability Kit**			BioLegend, San Diego, CA, USA	423102
**Zombie NIR Fixable Viability Kit**			BioLegend, San Diego, CA, USA	423105

Supplementary Table S2: Primers used for single-cell mRNA analysis:

Target	*Forward primer (5‘ to 3‘)*	*Reverse primer (5‘ to 3‘)*
**AHR**	TAGGCTCAGCGTCAGTTACC	TGGCCTCCGTTTCTTTCAGTA
**B2M**	TTAGCTGTGCTCGCGCTAC	TTAGCTGTGCTCGCGCTAC
**CCR5**	TGAGACATCCGTTCCCCTACA	TGGCAGGGCTCCGATGTATA
**CCR6**	AGGCAGCGATGTCTGTGAA	AGCTCAAGCCCCAACATCA
**CD38**	ACCTCACATGGTGTGGTGAA	GTTGCTGCAGTCCTTTCTCC
**CD69**	TCACCCATGGAAGTGGTCAA	ACACACTTGTCAGACCCTGTA
**CXCR3**	AACTGTGGCCGAGAAAGCA	TTGAGGCAGCAGTGCATGTA
**CXCR5**	ATCTTCTTCCTCTGCTGGTCAC	GGTATTGTCCACGGCCTTCA
**CXCR6**	TCTCTGGAACAAACTGGCAAA	CTGGCTGCTGTCATTGAAAC
**EOMES**	CTGTGGCAAAGCCGACAATA	CTCATCCAGTGGGAACCAGTA
**FCER1G**	CAGCAGTGGTCTTGCTCTTAC	GGAGGGTGAGGACAATTCCATA
**FCGR3A**	CATTCTTTCCACCTGGGTACCA	AGTCCTGTGTCCACTGCAAA
**GAPDH**	GAACGGGAAGCTTGTCATCAA	ATCGCCCCACTTGATTTTGG
**GZMK**	ATCCACAGTGGGTGCTGAC	AGAGTGTGCGCCTAAAACCA
**HLA-DRA**	CGCTCAGGAATCATGGGCTA	CGCCTGATTGGTCAGGATTCA
**IFNG**	ACTGCCAGGACCCATATGTAA	GTTCCATTATCCGCTACATCTGAA
**IL18**	ACCAAGGAAATCGGCCTCTA	ACCTCTAGGCTGGCTATCTTTA
**IL18R1**	GGGAGGCACAGACACCAAAA	TGAAGACGTGGCCTGGGATA
**IL22**	TATCACCAACCGCACCTTCA	GTTTCTCCCCAATGAGACGAAC
**IL2RB**	CACCGCCCCATGTCTCA	CAGGGGCCGCCATTACA
**IL7R**	GGAGAAAGTGGCTATGCTCAA	CTGCGATCCATTCACTTCCA
**IRF1**	AACAAGGATGCCTGTTTGTTCC	TGGGATCTGGCTCCTTTTCC
**IRF8**	GGAGTCAGCTCCTTCCAGAC	CGCGTCGTAGGTGGTGTA
**ITGA1**	CGGTGGAAGACATGTTTGGATA	GCCAACTAACGGAGAACCAA
**ITGAM**	GCCGGTGAAATATGCTGTCTAC	GGTATTCTCTGAGGCCGTGAA
**KLRB1**	GGACATTCAACAGAGCAGGAA	CTCGGAGTTGCTGCCAATATA
**KLRC3**	CAGGCCTGTGCTTCAAAGAA	CACCAATCCATGAGGAAGGTAA
**NCAM1**	ATGCGACCATCCACCTCAAA	GTGACCTGCTCCTCTAATTCCA
**NCR1**	AACCATGCCTGGTCTTTCCC	TGCAAGGCTGGTGTTCTCAA
**PDK1**	ACCAAGACCTCGTGTTGAGAC	AAGACGTGATATGGGCAATCCA
**PRF1**	GTACAGCTTCAGCACTGACAC	CTGGGTGGAGGCGTTGAA
**PTPRC**	GTGGCTTAAACTCTTGGCATTT	GGGAAGGTGTTGGGCTTT
**RORA**	CAGCAGATAACGTGGCAGAC	GGCACACAATTGCCACATCA
**RPL13A**	GAGGCCCCTACCACTTCC	GCCGTCAAACACCTTGAGAC
**RUNX3**	AGCACCACAAGCCACTTCA	TCGGAGAATGGGTTCAGTTCC
**S1PR1**	TCATCGTCCGGCATTACAAC	ACACCACCGAGGTCAGTTTA
**S1PR5**	CGCCTTCATCGTGCTAGAGAA	GGAACATGGGAGCGTGGAA
**SERPINB1**	TGCCGGATGACATTGAGGAC	AGTCCACTCATGCAACTTTTCCA
**SMAD4**	CACCAAAACGGCCATCTTCA	GGAATGCAAGCTCATTGTGAAC
**SMAD7**	CTTCATCAAGTCCGCCACAC	TTGATGGAGAAACCGGGGAA
**STAT1**	ATGCTGGCACCAGAACGAA	GCTGGCACAATTGGGTTTCAA
**STAT3**	AGCATCGAGCAGCTGACTAC	CCATGTGATCTGACACCCTGAA
**STAT4**	GCAAGACGAATTTGACTACAGGTA	TCCTGATTCACCATGGCACTA
**STAT5A**	CCCAGGCTCCCTATAACATGTA	ATGGTCTCATCCAGGTCGAA
**STAT6**	TGTCCTGGTCGCAGTTCA	TCCAGGACACCATCAAACCA
**TBX21**	GGGCGTCCAACAATGTGAC	CCGTCGTTCACCTCAACGATA
**TGFBR1**	ACAGAAGTTAAGGCCAAATATCCC	GCTCCATTGGCATACCAACA

**TGFBR2**	TTCCCAGCTTCTGGCTCAAC	GGTCCCAGCACTCAGTCAAC
**TIGIT**	GTGGTGGTCGCGTTGACTA	TCCTGTCCAGCTGATTTTCTCC
**TNFSF10**	AGAAGGAAGGGCTTCAGTGAC	CCTGGACCTCCATCATAGCC
**ZBTB16**	AGCCACACGGCTCTCAAA	AGCTGCCACAGAACTCACA
**ZEB2**	AGGCCAATGGGCAAGAAGAA	AGGTCAGCAGTTGGGCAAA

Supplementary Table S3: Differentially expressed genes comparing patient derived ihCD56^{bright} NK cells *(liver)*, patient derived pbCD56^{bright} NK cells *(blood)* and healthy control pbCD56^{bright} NK cells *(blood control).*

Differentially expressed genes	Groups		
CCR5	Ref group (liver)	.	
	Group 2 (blood)	p<0.0001	***
	Group 3 (blood control)	p<0.0001	***
CCR6	Ref group (liver)	.	
	Group 2 (blood)	0.996001	
	Group 3 (blood control)	p<0.05	*
CD38	Ref group (liver)	.	
	Group 2 (blood)	p<0.0001	***
	Group 3 (blood control)	0.000052642	***
CD69	Ref group (liver)	.	
	Group 2 (blood)	p<0.0001	***
	Group 3 (blood control)	p<0.0001	***
CXCR3	Ref group (liver)	.	
	Group 2 (blood)	p<0.0001	***
	Group 3 (blood control)	p<0.0001	***
CXCR6	Ref group (liver)	.	
	Group 2 (blood)	p<0.0001	***
	Group 3 (blood control)	p<0.0001	***
EOMES	Ref group (liver)	.	
	Group 2 (blood)	p<0.0001	***
	Group 3 (blood control)	p<0.0001	***
FCER1G	Ref group (liver)	.	
	Group 2 (blood)	p<0.0001	***
	Group 3 (blood control)	p<0.0001	***
FCGR3A	Ref group (liver)	.	
	Group 2 (blood)	p<0.05	*
	Group 3 (blood control)	0.143934	
IFNG	Ref group (liver)	.	
	Group 2 (blood)	p<0.0001	***
	Group 3 (blood control)	p<0.0001	***
IL18	Ref group (liver)	.	
	Group 2 (blood)	p<0.0001	***
	Group 3 (blood control)	0.657329	
IL18R1	Ref group (liver)	.	
	Group 2 (blood)	p<0.0001	***
	Group 3 (blood control)	0.148225	
IL22	Ref group (liver)	.	
	Group 2 (blood)	p<0.0001	***
	Group 3 (blood control)	p<0.001	**
IL2RB	Ref group (liver)	.	
	Group 2 (blood)	p<0.0001	***
	Group 3 (blood control)	p<0.05	*

IL7R	Ref group (liver)	.	
	Group 2 (blood)	p<0.0001	***
	Group 3 (blood control)	0.0560555	
IRF1	Ref group (liver)	.	
	Group 2 (blood)	p<0.05	*
	Group 3 (blood control)	0.474638	
IRF8	Ref group (liver)	.	
	Group 2 (blood)	p<0.0001	***
	Group 3 (blood control)	p<0.0001	***
ITGA1	Ref group (liver)	.	
	Group 2 (blood)	p<0.05	*
	Group 3 (blood control)	0.20705	
ITGAM	Ref group (liver)	.	
	Group 2 (blood)	p<0.0001	***
	Group 3 (blood control)	p<0.05	*
KLRB1	Ref group (liver)	.	
	Group 2 (blood)	p<0.0001	***
	Group 3 (blood control)	p<0.0001	***
KLRC3	Ref group (liver)	.	
	Group 2 (blood)	0	***
	Group 3 (blood control)	0.959982	
NCAM1	Ref group (liver)	.	
	Group 2 (blood)	p<0.0001	***
	Group 3 (blood control)	0.971472	
NCR1	Ref group (liver)	.	
	Group 2 (blood)	p<0.001	**
	Group 3 (blood control)	p<0.0001	***
PRF1	Ref group (liver)	.	
	Group 2 (blood)	p<0.0001	***
	Group 3 (blood control)	0.648428	
PTPRC	Ref group (liver)	.	
	Group 2 (blood)	p<0.001	**
	Group 3 (blood control)	p<0.05	*
RORA	Ref group (liver)	.	
	Group 2 (blood)	p<0.0001	***
	Group 3 (blood control)	p<0.0001	***
RUNX3	Ref group (liver)	.	
	Group 2 (blood)	p<0.0001	***
	Group 3 (blood control)	0.640389	
S1PR1	Ref group (liver)	.	
	Group 2 (blood)	p<0.0001	***
	Group 3 (blood control)	p<0.001	**
S1PR5	Ref group (liver)	.	
	Group 2 (blood)	p<0.0001	***
	Group 3 (blood control)	p<0.0001	***
SERPINB1	Ref group (liver)	.	
	Group 2 (blood)	p<0.0001	***
	Group 3 (blood control)	p<0.05	*

STAT1	Ref group (liver)	.	
	Group 2 (blood)	p<0.0001	***
	Group 3 (blood control)	0.878365	
STAT3	Ref group (liver)	.	
	Group 2 (blood)	0.227315	
	Group 3 (blood control)	p<0.05	*
STAT4	Ref group (liver)	.	
	Group 2 (blood)	p<0.0001	***
	Group 3 (blood control)	p<0.0001	***
STAT6	Ref group (liver)	.	
	Group 2 (blood)	0.89722	
	Group 3 (blood control)	p<0.05	*
TBX21	Ref group (liver)	.	
	Group 2 (blood)	p<0.001	**
	Group 3 (blood control)	0.0931068	
TGFBR2	Ref group (liver)	.	
	Group 2 (blood)	p<0.0001	***
	Group 3 (blood control)	p<0.0001	***
TIGIT	Ref group (liver)	.	
	Group 2 (blood)	p<0.0001	***
	Group 3 (blood control)	p<0.0001	***
TNFSF10	Ref group (liver)	.	
	Group 2 (blood)	p<0.05	*
	Group 3 (blood control)	p<0.001	**
ZBTB16	Ref group (liver)	.	
	Group 2 (blood)	p<0.0001	***
	Group 3 (blood control)	p<0.0001	***
